# 

**Published:** 1904-09

**Authors:** 


September, 1904. VOL. XXV. No. 9.

### THE INTERNATIONAL DENTAL JOURNAL.

JAMES TRUMAN, D.D.S., EDITOR,
PHILADELPHIA.

*COLLABORATORS:*

WILLIAM H. POTTER, A.B., D.M.D., BOSTON.

CHARLES P. BRIGGS, A.B., M.D., D.M.D., BOSTON.

LIBRARY INDEXED S. G. O.

LIBRARY SURGEON GENERAL'S OFFICE OCT. 17-1904

### Summary of Contents.

(*For details, see p. 2.*)

ORIGINAL COMMUNICATIONS.

REPORTS OF SOCIETY MEETINGS.

DOMESTIC CORRESPONDENCE.

MISCELLANY.

EDITORIAL.

BIBLIOGRAPHY.

CURRENT NEWS.

OBITUARY.

REVIEWS OF DENTAL LITERATURE.

*$2.50 a Year, in Advance. Single Copies, 25 Cents.*

PUBLISHED MONTHLY BY

The International Dental Publication Company,

227–231 SOUTH SIXTH ST., PHILADELPHIA.

EDITORIAL OFFICE, 4505 CHESTER AVENUE, PHILADELPHIA, PA.

PRINTED BY J. B. LIPPINCOTT COMPANY, PHILADELPHIA.

Entered at the Post-Office at Philadelphia, and admitted for transmission through the mails at second-class rates.

